# Supplementary material for: Patterns of Intron Gain and Loss in Fungi
Source: PLoS Biol. 2004 Nov 30;2(12):e422. doi: 10.1371/journal.pbio.0020422 (PMC532390; doi:10.1371/journal.pbio.0020422)
Supplement: Table S1 — Also available at http://genes.mit.edu/NielsenEtAl/. (4.3 MB ZIP). [file pbio.0020422.st001.zip › NielsenEtAl/html/58.html]

AN1861.1.NCU00361.1.MG05180.1.FG06739.1


```
 CLUSTAL W (1.82) Multiple Sequence Alignments - Introns Inserted


Sequence 1: MG05180.1	340 aa
Sequence 2: FG06739.1	273 aa
Sequence 3: NCU00361.1	339 aa
Sequence 4: AN1861.1	297 aa
Alignment Length: 341 aa
Number Identitical Residues: 135 aa
Alignment Score (without introns) 6635


MG05180.1 	MQD1LSSLMRQDSGVITGNICGAAPLPSVDSPKATTFFGARASVNLR1DTKMSDSTPTEA
NCU00361.1	MPP~EKKRKTEPEDKAEVTQQENDVAESTTEPNNQTVTDSKEEPSVT~EAALATTSSSSP
FG06739.1 	---~-------------------------------------------~---MAPGTPTKD
AN1861.1  	---~---------------------MSSERDQEQLSLEESREGEGKS~TTSQTESNASDV
          	                           :  . .  :   :    .    :  :  ..:. 

MG05180.1 	AVAESNTAGNEATTNSDKASPETTTTSTTEPGNTAKTAAAERMARFRALQARA~KTSSDQ
NCU00361.1	PVLSASETAQPDTAATSQSSSTPPTSTSAAESAAAK--ARERAERFRALQARA~KSSSQQ
FG06739.1 	SPVEKTDD----------------TKQESAPDNVSK--AQDRMARFKALQARA~KTSSEK
AN1861.1  	AEDPSTKE----------------QTGDKEDSAAAR--ARERKERFKALQARA0KSATER
          	.    .                   .     . .::  * :*  **:****** *:::::

MG05180.1 	NLKAATAESRREASDPSQLTALHRRHAIASHKILKADVEESGGDFERKRAWDWTAEESER
NCU00361.1	NLKKATKESQRLQSDPSQLTALGRRHAIASHKLLKADIEDAGGDFERKRAWDWTVEESER
FG06739.1 	NLQEATLESQRLATDPSQLTAIHRKHAIASHKLLKADVEESGGDFERKRAWDWTVDESEK
AN1861.1  	NLKETAAETQRLATDPSLLSSLSRKHAFASHNLLKADTEAAGEDFERKRAWDWTVDESEK
          	**: :: *::*  :*** *::: *:**:***::**** * :* ***********.:***:

MG05180.1 	WDKRVKKKEAARDNNAFQDYRHEAEKIYKRQLKNMP-PDMERYAKDKMAAIDKAAAAGTL
NCU00361.1	WDKRMKKKEAHRDDTAFRDYNQQAEKSYKRQLRNMGGPDLERYTREKLAAIEKAAAAGTL
FG06739.1 	WDKRLKKKAARRDNNAFADFTQEANKIYKRQLKNID-MNMEKYEKDKQALIEKAAASGGL
AN1861.1  	WDRRMEKKQRHRDDVAFQDYTQDARKVYKRQLREMK-PDLEGYENEKMAAIEKAAASGDL
          	**:*::**   **: ** *: ::*.* *****:::   ::* * .:* * *:****:* *

MG05180.1 	DIVETEDGELIAVDRAGTFYSTTDSTDFAKNKPDKEAIDRLVNDMKKAEDVAAKKRKARM
NCU00361.1	EIVETEDGEMIAIDKDGTFFSTADSTQFAQHKPDKAAVDRLVADMRKAEEASLKKRRERM
FG06739.1 	DIVETEDGELIAVDKDGSFYSTADSTTFAQNKPDKTAVDRLVADLQRAEEQRLKKRKERM
AN1861.1  	EIVETNDGEMIAVDKNGTFYSTADTIGFTESKPDRAAVDKLVADLRKAEEVRLKKRRDRR
          	:****:***:**:*: *:*:**:*:  *:: ***: *:*:** *:::**:   ***: * 

MG05180.1 	AKNGEDDDVTYINDKNKQFNQKLARFYNK~YTAEIRESFERGTMI
NCU00361.1	ANNGDDADVTYINEKNKQFNQKLSRFYNK~YTAEIRDSFERGTMV
FG06739.1 	AQNGDDGDVTYINEKNKQFNQKLSRFYNQ~YTAEIRDSFERGTMI
AN1861.1  	GGD-EDGDVTYINEKNKQFNQKLARFYNK0YTTEIRDSFERGTMI
          	. : :* ******:*********:****: **:***:*******:
```
